# A Clinico-Epidemiological Study of Hyperkeratotic Palmoplantar Lesions and Its Correlation With Dermoscopy and Histopathology in a Tertiary Care Center

**DOI:** 10.7759/cureus.36561

**Published:** 2023-03-23

**Authors:** Jahnavi Chaduvula, Dilipchandra Chintada, Jami Vijayashree, Sai Sriya Chalamalasetty, Kirankanth Vudayana, Anandkumar Vaggu

**Affiliations:** 1 Department of Dermatology, Great Eastern Medical School and Hospital, Srikakulam, IND

**Keywords:** dermoscopy of dermatophytosis, dermoscopy of palmoplantar lichen planus, dermoscopy of hyperkeratotic eczema, dermoscopy of palmoplantar psoriasis, palmoplantar hyperkeratosis

## Abstract

Introduction

Hyperkeratotic lesions on the palms and soles are one of the most frequent clinical presentations encountered in dermatological practice, with a myriad of underlying etiologies that closely resemble one another and are clinically indistinguishable. Histopathological examination is the tool used by dermatologists to arrive at a final diagnosis, but it is invasive and not feasible under all circumstances. Dermoscopy is a new age, increasingly popular, noninvasive diagnostic technique of great value that is used to diagnose underlying etiology by acting as a bridge between clinical and histopathological pictures. This study aimed to evaluate the various etiologies underlying palmoplantar hyperkeratosis and the role of dermoscopy in the diagnosis of each disease along with its ability to delineate a close differential diagnosis and ensure appropriate treatment.

Materials and methods

This was a hospital-based observational cross-sectional study conducted from July 1 to December 31, 2022. Consenting patients with hyperkeratotic palmoplantar lesions on clinical examination attending the dermatology outpatient department at our tertiary care hospital were included after institutional ethical clearance was obtained. Patients with HIV, hepatitis B surface antigen (HBsAg), hepatitis C virus (HCV) infection, or a history of hyperkeratotic lesions since birth, i.e., inherited palmoplantar keratodermas, were excluded from the study. A total of 60 patients aged between 18 and 60 years who met the above criteria were included. A complete history was taken; a thorough examination was performed. Routine investigations and tissue histology were done. Potassium hydroxide (KOH) mount and patch testing were done as and when required. Dermoscopy with DermLite DL4 was performed in all cases on lesional areas, and the findings were noted.

Results

Palmoplantar psoriasis has been found to be the most common cause of hyperkeratosis in our study with 24 (40%) out of 60 cases, followed by chronic hand-foot eczema found in 19 (31%) cases. Dermoscopic findings that help in differentiating various etiologies are vascular findings and scaling types. Vascular findings, mainly regularly arranged dots and globules, were more prominent in palmoplantar psoriasis. Yellow white scaling was frequently observed in hyperkeratotic hand eczema. Most of the cases corresponded with their provisional diagnoses on histopathology, but four out of 19 histopathologically confirmed cases of eczema showed clinical resemblance to palmoplantar psoriasis, along with dermoscopic features of psoriasis. Two out of four cases of histopathologically confirmed palmoplantar LP were clinically considered palmoplantar psoriasis and hyperkeratotic hand-foot eczema.

Conclusion

Although hyperkeratoses of palms and soles are a common clinical entity, the similarity between the clinical features of the underlying conditions causes a diagnostic dilemma for treating dermatologists. Dermoscopy is a noninvasive, quick, reproducible, supportive investigation in the diagnosis of these conditions that certainly aids in reaching closer to a differential diagnosis and for better delineation, but it does not avert the need for a skin biopsy. Further confirmation with histopathological examination is advisable, especially in these conditions as they show close morphological similarity. A combination of all these investigations and clinical examinations gives better diagnoses and appropriate treatment.

## Introduction

Hyperkeratotic lesions on the palms and soles are one of the most frequent clinical presentations encountered in dermatological practice, with a myriad of underlying etiologies that closely resemble one another and are clinically indistinguishable. Histopathological examination is the tool used by dermatologists to arrive at a final diagnosis. However, it is invasive and not feasible under all circumstances. Dermoscopy is a new age, increasingly popular, noninvasive diagnostic technique of great value that is used to diagnose underlying etiology by acting as a bridge between clinical and histopathological pictures. This study aimed to evaluate the various etiologies underlying palmoplantar hyperkeratosis and the role of dermoscopy in the diagnosis of each disease along with its ability to delineate a close differential diagnosis and ensure appropriate treatment.

## Materials and methods

This was a hospital-based observational cross-sectional study that was conducted from July 1 to December 31, 2022. Consenting patients with hyperkeratotic palmoplantar lesions on clinical examination, who have not received prior treatment, topical or systemic, attending the dermatology outpatient department at our tertiary care hospital were included after institutional ethical clearance was obtained. Patients with HIV, hepatitis B surface antigen (HBsAg), hepatitis C virus (HCV) infection, or a history of hyperkeratotic lesions since birth, i.e., inherited palmoplantar keratodermas, were excluded from the study. Inherited palmoplantar keratodermas are a rare heterogenous group of disorders that occur due to mutations in genes encoding keratinocyte formation and differentiation. They present with distinct clinical patterns such as punctate, papular, waxy, striate keratodermas as an isolated symptom or as a part of a syndrome with systemic abnormalities, based on which these cases were excluded.

A total of 60 patients aged between 18 and 60 years who met the above criteria were included in this study. A complete history was taken; thorough general, systemic, and cutaneous examinations were performed; and clinical findings were noted. Routine investigations such as complete blood picture, liver function tests, renal function tests, viral markers, random blood sugar, and tissue histology were done. Potassium hydroxide (KOH) mount and patch testing were done as and when required. Scrapings were taken from at least two locations, preferably one palm and sole if both palms and soles were involved, to detect fungal elements in cases where dermatophytosis was suspected.

Dermoscopy with DermLite DL4 and in both polarized and non-polarized modes was performed in all cases on lesional areas, and the findings were noted.

## Results

Clinical findings

Of the 60 patients included in this study, 31 were females and 29 were males. Peak incidence was seen in the 35-50 age group. Lesions involved both palms and soles in 33 (55%) patients; they involved the soles alone in 17 (28%) patients, the palms alone in eight (12%) patients, and both soles and one palm in three (5%) patients (Table 1). Itching was the most common associated symptom, present in 43 (71%) patients; fissuring was seen in 36 (60%) patients, pain in 39 (65%), and burning sensation in 33 (55%). Twenty (33%) patients were asymptomatic (Table 2). The mean duration of symptoms was 4.5 years for palmoplantar psoriasis, 1.5 years for eczema, four months for dermatophytosis, and two years for palmoplantar lichen planus (LP).

**Table 1:** Distribution of hyperkeratotic lesions over the palms and soles Bilateral symmetry is most frequently noted in psoriasis and acquired palmoplantar keratoderma. Tinea: dermatophytosis, PPK: palmoplantar keratoderma, LP: lichen planus

Diagnoses	Bilateral		Unilateral
	Symmetrical	Asymmetrical	
Psoriasis	18 (75%)	4 (16%)	2 (8.3%)
Eczema	7 (36.8%)	9 (47%)	3 (15.7%)
PPK	4 (44.4%)	4 (44.4%)	1 (11.1%)
Tinea	1 (25%)	1 (25%)	2 (50%)
LP	1 (25%)	3 (75%)	0 (0%)

**Table 2:** Symptoms coexisting with hyperkeratosis Itching is the most common symptom associated with eczema, followed by tinea and palmoplantar LP. Fissuring is the most common symptom coexisting with psoriasis. Tinea: dermatophytosis, PPK: palmoplantar keratoderma, PPLP: palmoplantar lichen planus

Symptoms	Psoriasis	Eczema	Tinea	PPLP	PPK
Itching	15 (62%)	17 (89%)	3 (75%)	3 (75%)	5 (55%)
Fissuring	20 (83%)	9 (47%)	1 (25%)	2 (50%)	4 (44%)
Pain	18 (75%)	11 (57%)	1 (25%)	2 (50%)	7 (78%)
Burning sensation	10 (41%)	13 (68%)	0 (0%)	3 (75%)	7 (78%)
Asymptomatic	11 (45%)	4 (21%)	1 (25%)	1 (25%)	3 (33%)

Clinically similar cutaneous lesions over other sites, suggestive of an underlying etiology, were present in 10 of 24 cases of palmoplantar psoriasis, six of 19 cases of eczema, three of four cases of tinea, and three of four cases of LP. Associated mucosal or nail findings were present in 15 of the 24 cases of palmoplantar psoriasis, nine of the 19 cases of hyperkeratotic hand and foot eczema, two of the four cases of palmoplantar lichen planus, and three of the nine cases of palmoplantar keratoderma.

**Figure 1:**
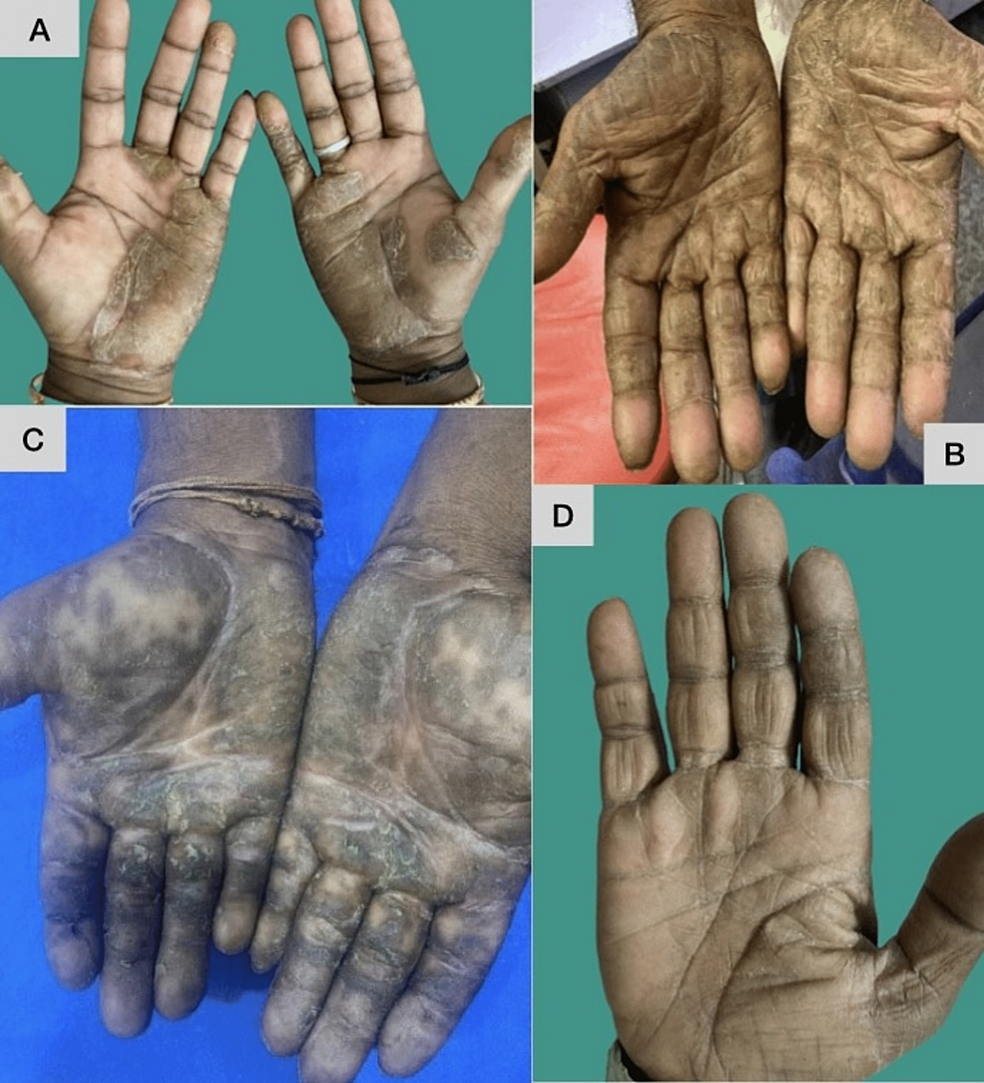
Clinical presentations of histopathologically confirmed palmoplantar psoriasis, hyperkeratotic hand eczema, palmoplantar lichen planus, and tinea manuum A: Clinical picture of palmoplantar psoriasis, B: clinical picture of hyperkeratotic hand eczema, C: clinical picture of palmoplantar lichen planus, D: clinical picture of tinea manuum

Palmoplantar psoriasis (Figure 1A) was the most common cause of hyperkeratosis in our study, found in 24 (40%) of 60 patients. Chronic hand and foot eczema (Figure 1B) was the second most common cause, found in 19 (31%) cases. Acquired palmoplantar keratoderma, hyperkeratotic tinea pedis and tinea manuum (Figure 1D), and palmoplantar LP (Figure 1C) were the other etiologies found in nine (15%), four (6%), and four (6%) patients, respectively (Table 3).

**Table 3:** Number of cases diagnosed with each condition The disease with the highest number of cases diagnosed was psoriasis, with an equal distribution among males and females. Tinea: dermatophytosis, PPK: palmoplantar keratoderma, LP: lichen planus

Diagnosis	Total	Males	Females
Psoriasis	24	12	12
Eczema	19	9	10
Tinea	4	1	3
LP	4	2	2
PPK	9	5	4

KOH mount was negative in two of four patients diagnosed with dermatophytosis. Periodic acid-Schiff (PAS) positivity on histopathology was noted in three of the four patients.

Patch testing was positive in six of the 19 cases of chronic hand and foot eczema. All six cases were also positive for potassium dichromate. The onset of lesions after contact with fertilizers was observed in five of nine patients diagnosed with acquired palmoplantar keratoderma. The onset of lesions secondary to longstanding Hansen’s disease despite treatment was noted in two of these five patients.

Dermoscopic findings

**Figure 2:**
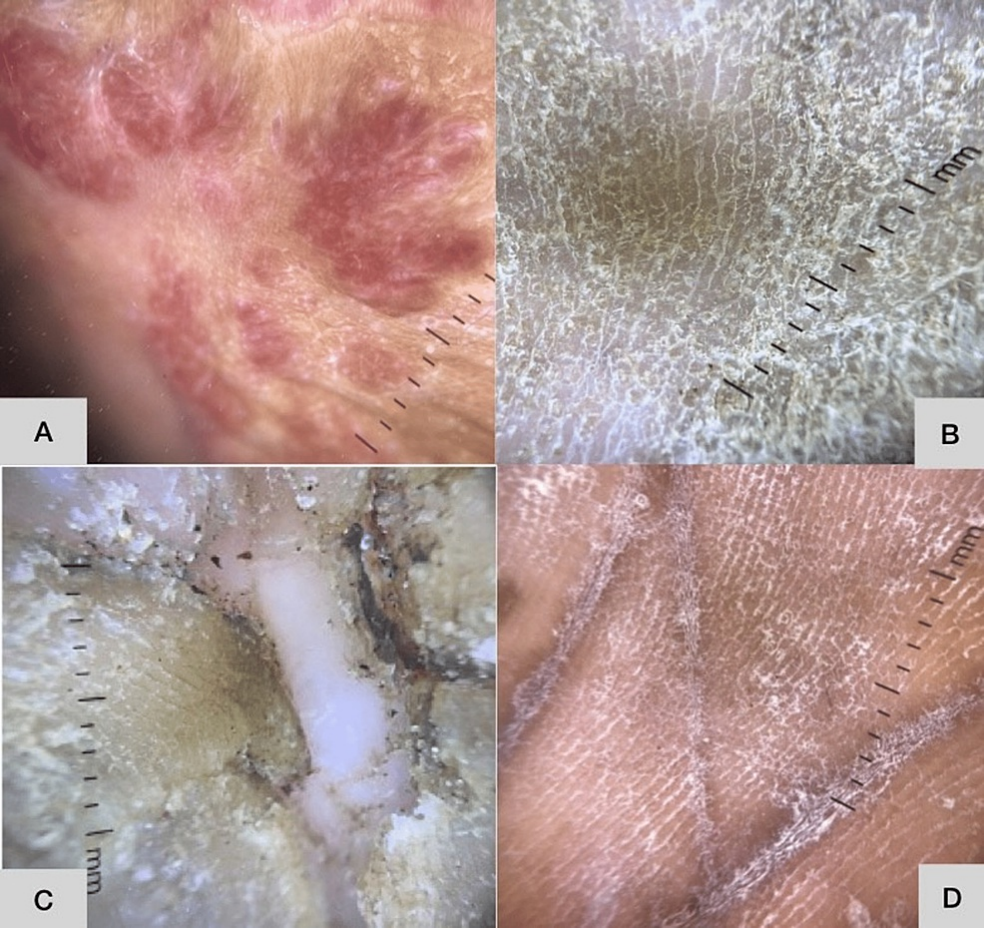
Dermoscopy of palmoplantar psoriasis, hyperkeratotic hand eczema, palmoplantar lichen planus, and tinea pedis Vascularity in palmoplantar psoriasis is visualized as regularly arranged red dots and globules. Yellow-white scaling is seen in hyperkeratotic hand and foot eczema. Concentric scaling is seen in palmoplantar lichen planus. White powdery scaling along dermatoglyphics is noted in dermatophytosis. A: Dermoscopy of palmoplantar psoriasis, B: dermoscopy of hyperkeratotic hand eczema, C: dermoscopy of palmoplantar lichen planus, D: dermoscopy of tinea pedis

**Figure 3:**
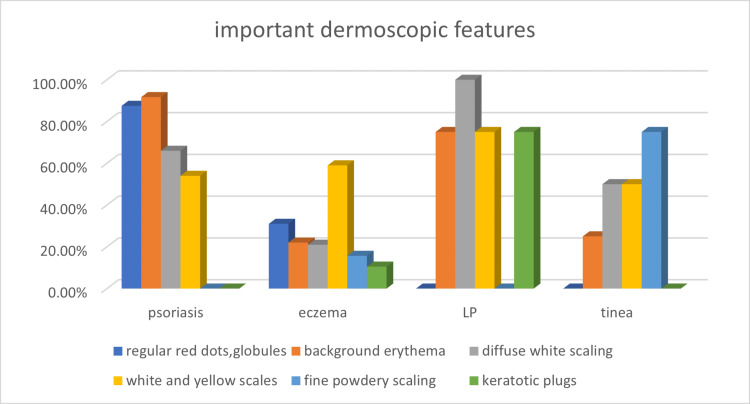
Important features of psoriasis, eczema, LP, and tinea observed on dermoscopy The percentages of cases showing the dermoscopic features in each disease are plotted on the y-axis, and the diseases are plotted on the x-axis. Tinea: dermatophytosis, tinea manuum, tinea pedis, LP: palmoplantar lichen planus

On dermoscopy in this study, the most common recurrent features of palmoplantar psoriasis were diffuse scaling in 20 (83%) patients and regularly spaced red dots and occasional globules over a light red background in 21 (87.5%). The various colors of scaling included white (66%), white and yellow (54%), greasy yellow (41%), and a combination of all three colors (36%). Vascular features included regular red dots and globules in 87.5% of patients (this was the most common) and irregular red dots and globules in 75%. Differentiated vessels including branched vessels, such as arborizing and glomerular vessels, and linear vessels were found in 7.5% and 62.5% of patients, respectively. Undifferentiated vessels, i.e., vascular structures that do not conform to a particular morphology, were also noted in 32% of patients (Figures 2A, 3 and Tables 4, 5).

**Table 4:** Major dermoscopic features of psoriasis and eczema observed in the participants of this study Vascularity is a prominent feature of psoriasis, while scaling is a prominent feature of eczema.

Dermoscopic features	Psoriasis	Eczema
Scaling		
White scales	16 (66%)	4 (21%)
Yellow white scales	13 (54%)	11 (59%)
Yellow scales	10 (41%)	6 (31%)
Vascularity		
Regular red dots and globules	21 (87.5%)	6 (31%)
Irregular red dots and globules	1 (4.5%)	10 (52%)
Arborizing, glomerular vessels	9 (37.5%)	3 (15.7%)
Linear vessels	15 (62.5%)	5 (26.3%)
Undifferentiated vessels	8 (32%)	3 (15.7%)

**Table 5:** Major dermoscopic features of psoriasis, eczema, palmoplantar LP, and tinea Background erythema and scaling are the most common findings among all patients. Tinea: dermatophytosis, LP: lichen planus

Dermoscopic features	Psoriasis	Eczema	LP	Tinea
Regular red dots, globules	87.50%	31%	0%	0%
Background erythema	91.70%	22%	75%	25%
Diffuse white scaling	66%	21%	100%	50%
White and yellow scales	54%	59%	75%	50%
Fine powdery scaling	0%	15.70%	0%	75%
Keratotic plugs	0%	10.50%	75%	0%

In hyperkeratotic hand eczema, diffusely distributed white and yellow scaling with dotted-type vessels in a patchy, irregular distribution (Table 5) over a yellowish-dull red background were noted the most. Greasy, dull yellow scaling was the most prevalent finding in 59% of cases (Figure 2B); additional features of brownish‑orange dots/globules and yellow‑orange clods were observed in 65% of cases. Vascular structures were less prominent in hyperkeratotic hand eczema than in psoriasis (Figure 3). They were noted in the following order: as red dots and globules in irregular, patchy distributions in 52% of cases, in a regular distribution in 31%, as linear vessels in 26.3%, and as undifferentiated and differentiated vessels in 15.7% each (Table 4).

Dermoscopy in three of the four patients with dermatophytosis, tinea manuum, and tinea pedis showed a fine white powdery scaling in the dermatoglyphics of interdigital areas (Figure 2D), with apparent sparing of the skin in between the dermatoglyphic lines (Table 5). A faint background erythema was observed in one of the four patients, and diffuse white scaling and a combination of yellow and white scales were found in two of the four cases (Figure 3).

Diffuse white scaling was the most prominent feature of palmoplantar LP, found in all four of the patients diagnosed (Figure 3); it was followed by yellow and white scaling, which was found in three of the four cases (Figure 2C). Background erythema and central keratotic plugs surrounded by collarette and concentric scaling were observed in three of the four cases (Table 5).

Histopathological findings

**Figure 4:**
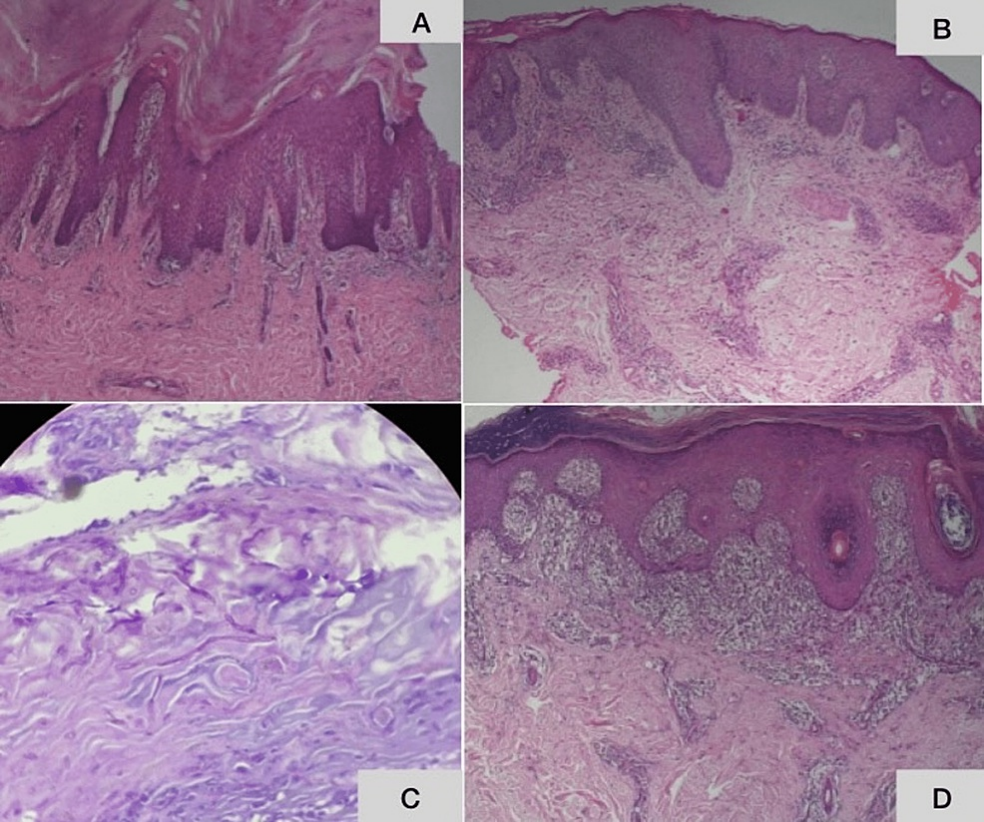
Histopathology of palmoplantar psoriasis, hyperkeratotic hand and foot eczema, tinea, and palmoplantar lichen planus Histopathology of palmoplantar psoriasis showing compact hyperkeratosis with foci of parakeratosis. Histopathology of hyperkeratotic hand and foot eczema showing spongiotic changes. Histopathology of tinea showing PAS positivity for fungal elements. Histopathology of palmoplantar lichen planus showing hyperkeratosis with lichenoid lymphocytic infiltrate. A: Histopathology of palmoplantar psoriasis, B: histopathology of hyperkeratotic hand eczema, C: histopathology of tinea pedis, D: histopathology of palmoplantar lichen planus PAS: periodic acid-Schiff

Most of the clinical presentations corresponded with their provisional diagnoses on histopathology. The characteristic features of psoriasis such as dilated and tortuous vessels and elongation of the rete ridges were seen in all 24 (100%) cases of psoriasis. Hyperkeratosis with foci of parakeratoses was observed in 22 of the 24 (91.6%) patients with psoriasis (Figure 4A), while suprapapillary thinning was observed in 21 of the 24 patients. Only 18 cases were clinically diagnosed as psoriasis.

Edematous changes in the papillary dermis along with spongiotic changes in the epidermis were observed in 17 of the 19 (89%) cases of hyperkeratotic hand eczema (Figure 4B). Additional psoriasiform hyperplasia was observed in six (31%) of the 19 cases; papillomatosis was seen in 18 of the 19 cases of hyperkeratotic eczema, and seven of these 19 cases had been clinically diagnosed as either psoriasis or palmoplantar keratoderma.

Lichenoid lymphocytic infiltrates with characteristic wedge-shaped hypergranulosis were observed in two (50%) of the four cases of palmoplantar LP. Two of the four cases also showed regular hypergranulosis with irregular epidermal hyperplasia and lichenoid infiltrates (Figure 4D). Parakeratosis was found in one of the four cases, and one of the four cases had been clinically diagnosed as hyperkeratotic hand eczema.

Hyperkeratosis and acanthosis were found in all four cases of dermatophytosis. PAS stain showed positivity in three of the four cases (Figure 4C), while focal neutrophilic spongiosis with focal parakeratosis was found in one of the four cases.

Moderate to severe hyperkeratosis was noted in 70.8% of the cases of psoriasis, 42% of the cases of eczema, and 50% of the cases of palmoplantar LP. Hyperkeratosis was relatively mild in dermatophytosis.

## Discussion

Clinical findings

In this study, a slight female preponderance was noted, with 51.6% of the study population being females. This differs from the studies conducted by Venkatesan et al. [1] and Gutte and Khopkar [2], in which males are more affected by palmoplantar dermatosis than females with a ratio of about 5:4. A similar predominance of the disease in males than in females was observed in other studies by Khandpur et al. [3] and Rathoriya [4].

In the present study, the incidence of palmoplantar LP was equal in males and females, which is dissimilar to the findings of Gutte and Khopkar [2], Sánchez-Pérez et al. [5], and Mugoni et al. [6], where female predominance was noted.

In the present study, itching was the predominant symptom, present in 43 (71%) of the 60 patients, irrespective of the diagnosis. This value is slightly lower than that found in a study conducted by Vijay Sekhar et al. [7], where itching was the predominant symptom in 64 (80%) of 80 patients, but similar to the findings of the study by Kumar et al. [8].

**Figure 5:**
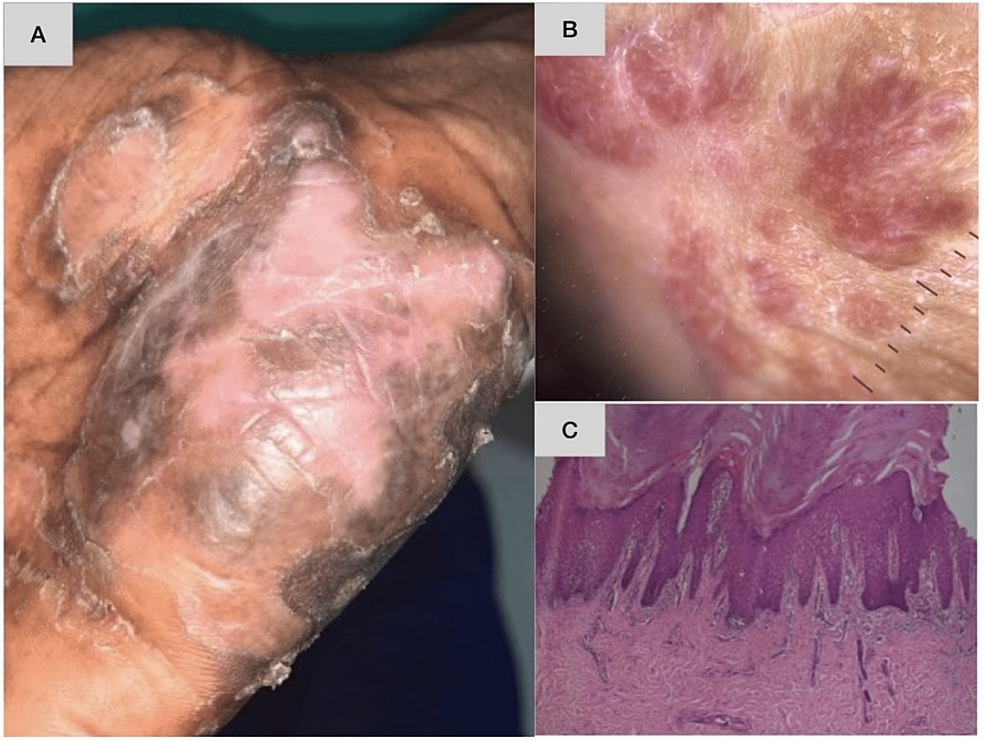
A case of palmoplantar psoriasis showing unilateral involvement with fissuring and hyperkeratosis Dermoscopy shows red dots, globules in a regular arrangement, and background erythema. Histopathology shows compact hyperkeratosis with foci of parakeratosis. A: Clinical picture, B: dermoscopic picture, C: histopathological picture

In the present study, lesions were bilateral in 52 of the 60 (86%) patients, which is lower than those in the study conducted by Vijay Sekhar et al. [7], where bilateral lesions were seen in 76 of 80 (92%) cases. Bilateral symmetry was most common in cases of palmoplantar psoriasis (Figure 5A), found in 75% of patients in the present study. A similar trend was observed in the study by Vijay Sekhar et al. [7], where more than 75% of cases of palmoplantar psoriasis showed bilateral lesions.

**Figure 6:**
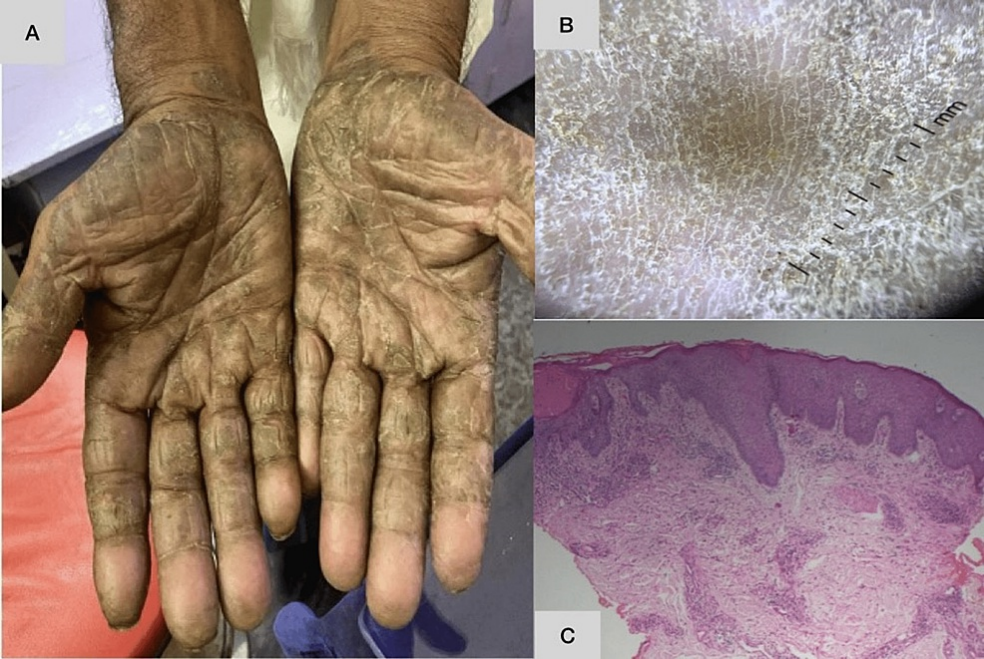
A case of chronic hand and foot eczema with bilateral symmetry showing yellow white greasy scaling with background erythema on dermoscopy Histopathology shows spongiotic changes along with foci of parakeratosis. A: Clinical picture, B: dermoscopic picture, C: histopathological picture

**Figure 7:**
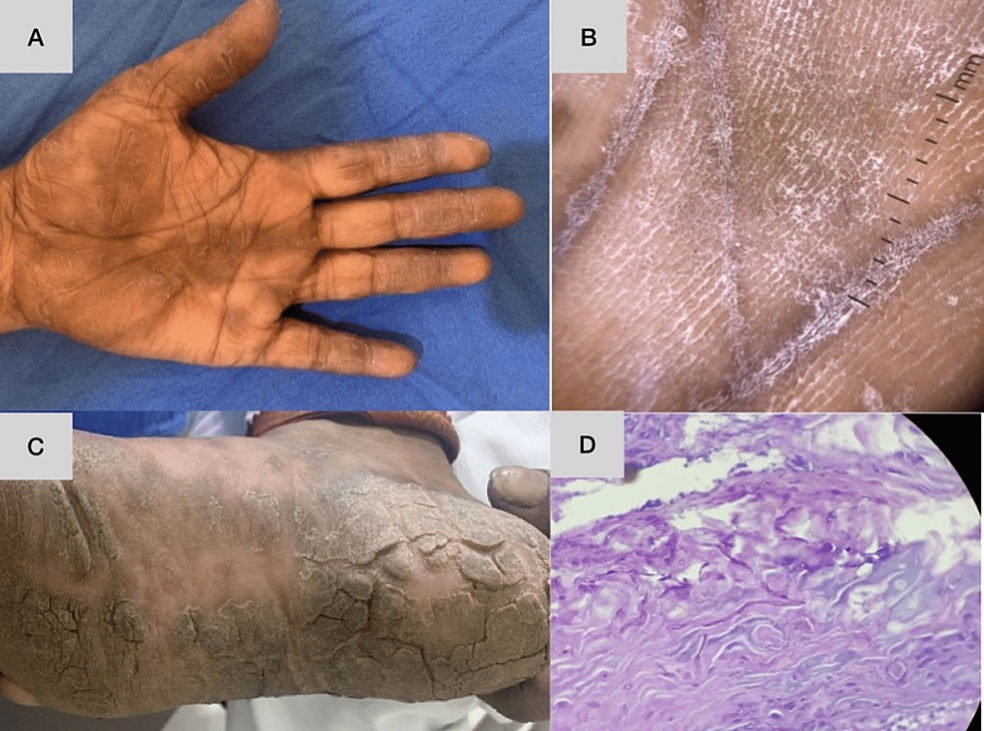
A case of tinea manuum and tinea pedis showing involvement of one hand and foot Dermoscopy of hand lesions shows diffuse white and yellow scaling more prominent over dermatoglyphics. Biopsy revealed hyperkeratosis with PAS positivity for fungal elements. A,C: Clinical picture, B: dermoscopic picture, D: histopathological picture PAS: periodic acid-Schiff

**Figure 8:**
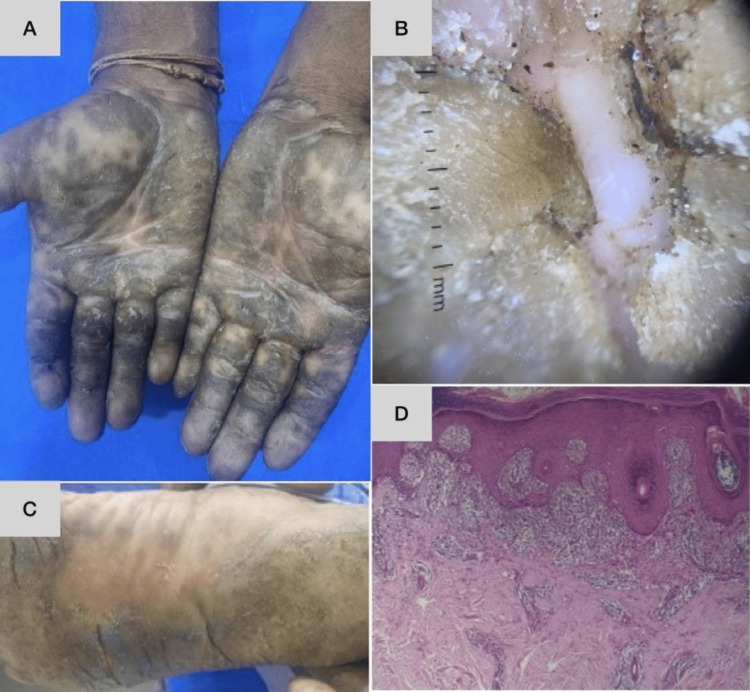
A case of palmoplantar lichen planus involving both hands and feet and showing symmetry Dermoscopy shows diffuse, concentric, yellow-white scaling. Biopsy shows hyperkeratosis along with wedge-shaped hypergranulosis and band-like lichenoid infiltrate. A,C: Clinical picture, B: dermoscopic picture, D: histopathological picture

In the present study, palmoplantar psoriasis was found to cause hyperkeratotic lesions of the hands and feet in 40% of patients (Figure 5A), hyperkeratotic hand and foot eczema in 31% (Figure 6A), acquired palmoplantar keratoderma in 15%, dermatophytosis in 6% (Figure 7A), and palmoplantar LP in 6% (Figure 8A, 8C). These findings are in concordance with those of the study by Vijay Sekhar et al. [7], where a diagnosis of psoriasis was made in 32 (40%) out of 80 patients and that of various types of eczema was made in 30 (37.5%). The number of cases diagnosed as fungal infections (tinea manuum, tinea pedis, or both) was 10 (12.5%); this is slightly greater than that in the present study, in which there were four (6%) out of 60. In the study by Kodali [9], 52% of cases were diagnosed as psoriasis; this is slightly higher than the number of cases of psoriasis in the present study. In the same study, 31% of patients were diagnosed with eczema and 2% with fungal infection, which is almost identical to the trends observed in the present study.

Dermoscopic and histopathological findings

Palmoplantar Psoriasis

In the current study, diffuse white scaling was the most prominent feature of psoriasis; it was observed in 83% of cases, which is almost identical to the findings of the studies by Adabala et al. [10] and Çetinarslan et al. [11], where predominantly diffuse scaling was observed in 76.3% (29/38) and 74.3% (26/35) of cases, respectively. Similar findings were also reported in the study conducted by Dassouli et al. [12].

In the present study, regularly spaced red dots and occasional globules over a light red background were the most consistent vascular finding in 87.5% (21 out of 24) of cases (Figure 5B); this is quite similar to a report on 14 cases of palmar psoriasis by Lallas et al. [13], where regularly distributed red vessels were reported in 77.3% of cases, and a report by Adabala et al. [10], where the dotted type was the most common vessel type in palmar psoriasis. Differentiated vessels were common in psoriasis, with linear vessels observed in 15 (62.5%) cases and arborizing and glomerular vessels observed in nine (37.5%) cases. Undifferentiated vessels were found in eight (32%) cases. In a study conducted by Adabala et al. [10], the glomeruloid variant of undifferentiated vessels was observed in 15.7% (6/38) of patients with psoriasis on the palm, following the observation that undifferentiated vessels were seen in 7.89% (3/38) of palmar psoriasis; this is almost identical to the findings in the present study.

In the present study, dilated and tortuous vessels were seen in all 24 (100%) cases of psoriasis; this is higher than the results of the study by Adabala et al. [10], where these vessels were noted in 87.5% (7/8) of cases. Elongation of rete ridges was observed in 99% of cases in the study by Kolesnik et al. [14], similar to the present study where it was found in 100% of cases (Figure 5C).

Hyperkeratotic Hand and Foot Eczema

In the present study, diffuse white and yellow scaling was noted in 54% of cases of hyperkeratotic hand and foot eczema (Figure 6B), which is close to the 56.4% reported by Çetinarslan et al. [11] and slightly lower than the 78.5% (11/14) reported by Adabala et al. [10].

In the present study, red dots and globules in irregular, patchy distribution were the most common finding in hyperkeratotic hand and foot eczema, observed in 52% of cases; this is in concordance with the findings of Çetinarslan et al. [11], where similar features were noted in 51.4% of patients with hyperkeratotic hand eczema. It is significantly lower than the finding in the study by Lallas et al. [13], where it was seen in 68.2% of cases.

On histopathological examination, hyperkeratosis was a constant feature in all cases of hyperkeratotic hand and foot eczema in the present study, similar to the findings by Agarwal et al. [15]. Spongiotic changes of the epidermis were seen in 17 of the 19 (89%) cases of hyperkeratotic hand eczema in the present study (Figure 6C), which is significantly higher than the 36% reported by Kolesnik et al. [14].

Dermatophytosis

In the present study, diffuse powdery white scaling was found in 75% of cases (Figure 7B), which is identical to the findings of the study by Manoharan et al. [16], where the most common dermoscopic finding in tinea pedis was white fine powdery scaling along the dermatoglyphics and adjoining furrows, with apparent sparing of the skin in between. Errichetti et al. [17] conducted a study where similar observations were mentioned in tinea manuum.

Hyperkeratosis and acanthosis were the most consistent findings; they were found in all four cases of dermatophytosis, similar to the study by Agarwal et al. [15], where they were found in 100% of cases. PAS positivity was noted in 75% of cases in our study (Figure 7C), similar to findings by Agarwal et al. [15], who reported findings of 73%.

Lichen Planus

Lichenoid lymphocytic infiltrates with characteristic wedge-shaped hypergranulosis were observed in two (50%) of the four cases of palmoplantar LP (Figure 8D) in this study; this is similar to the findings of the study conducted by Gutte et al. [2], in which palmoplantar LP showed characteristic histopathology very similar to that described for other sites in nearly 90% of cases. Parakeratosis was seen in 44% of cases in the aforementioned study, whereas it was only noted in 25% of cases in the current study.

**Figure 9:**
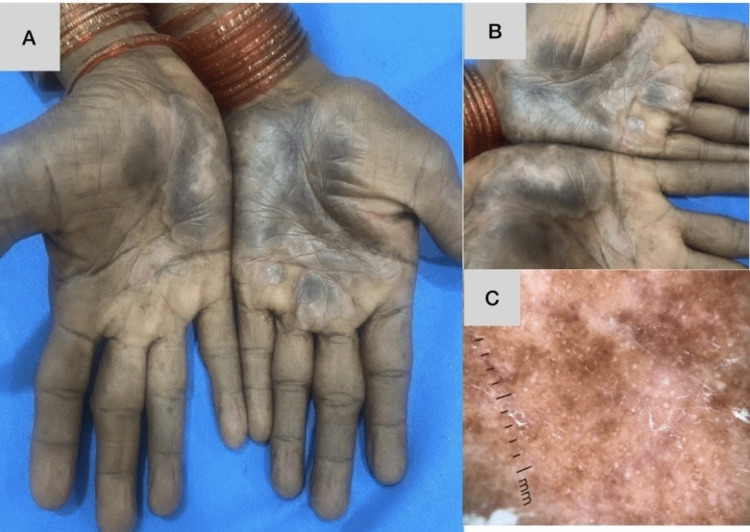
A case of histopathologically confirmed eczema resembling palmoplantar psoriasis clinically and dermoscopically Lesions are bilaterally symmetrical, with less obvious scaling and differentiated vascular structures including linear and branched vessels suggestive of psoriasis. A: Clinical picture, B: dermoscopic picture, C: histopathological picture

**Figure 10:**
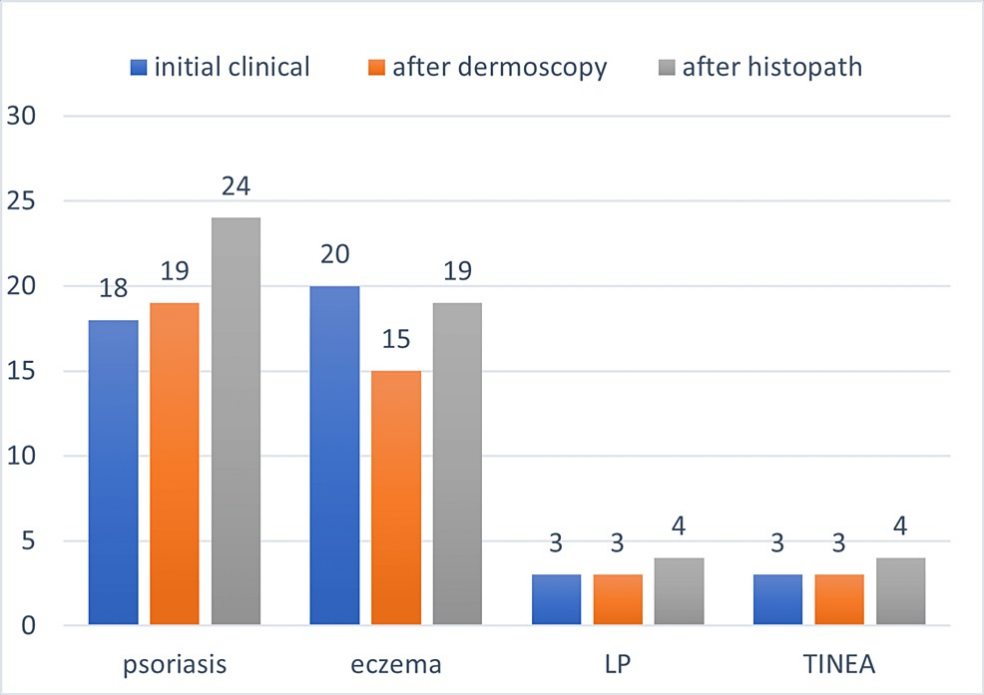
Final diagnoses after clinical, dermoscopic, and histopathological examination There is a considerable gap between the initial clinical diagnoses of psoriasis, eczema, LP, and tinea and the final histopathological diagnoses. Tinea: dermatophytosis, LP: lichen planus

In our study, four of the 19 cases of histopathologically confirmed hyperkeratotic hand and foot eczema showed clinical resemblance to palmoplantar psoriasis, along with dermoscopic features of psoriasis (Figure 9). Two of the four cases of palmoplantar LP were clinically diagnosed as either palmoplantar psoriasis or hyperkeratotic hand and foot eczema. There is considerable overlap between the clinical and dermoscopic features of the etiologies of hyperkeratotic lesions of the palms and soles (Figure 10).

## Conclusions

Although hyperkeratosis of the palms and soles is a common clinical entity, the similarity between the clinical features of the underlying conditions makes diagnosis a conundrum for the dermatologist. Most of these conditions are chronic and associated with occupational exacerbation. In these dermatoses, associated classical cutaneous features and involvement of other sites provide a clue for clinical diagnosis in some cases. There is a considerable difference in the treatment of these conditions, and inaccurate treatment can cause substantial psychological trauma to the patient and may be often interpreted as recalcitrance to treatment.

The aim of our study was to explore the extent to which dermoscopy was useful to arrive at the final diagnosis. Dermoscopy is a noninvasive, less time-consuming, reproducible, supportive investigation in the diagnosis of the underlying disorders that certainly aids in the making of a differential diagnosis and better delineates the conditions, but it does not obviate the need for histopathology. Further confirmation with histopathological examination is advisable, especially in the conditions discussed in this paper, since they show close morphological similarity. A combination of all these investigations and clinical findings aids in better diagnosis.
